# Variations in Functional and Anatomical Outcomes and in Proliferative Vitreoretinopathy Rate along a Prospective Collaborative Study on Primary Rhegmatogenous Retinal Detachments: The Retina 1 Project—Report 4

**DOI:** 10.5402/2012/206385

**Published:** 2012-10-08

**Authors:** J. Carlos Pastor, Itziar Fernández, Rosa M. Coco, María R. Sanabria, Enrique Rodríguez de la Rúa, Rosa M. Piñon, Vicente Martinez, Anna Sala-Puigdollers, José M. Gallardo, Sara Velilla

**Affiliations:** ^1^Institute of Applied Ophthalmobiology (IOBA), University of Valladolid, 47011 Valladolid, Spain; ^2^Department of Ophthalmology, Hospital Clínico Universitario de Valladolid, 47005 Valladolid, Spain; ^3^Ciber BBN, 50018 Zaragoza, Spain; ^4^Department of Ophthalmology, Complejo Asistencial de Palencia (CAPA), 34005 Palencia, Spain; ^5^Department of Ophthalmology, Hospital Universitario Puerta del Mar, 11009 Cádiz, Spain; ^6^Department of Ophthalmology, Hospital General Universitario de Burgos, 09005 Burgos, Spain; ^7^Department of Ophthalmology, Hospital Vall d'Hebrón, 08035 Barcelona, Spain; ^8^Department of Ophthalmology, Hospital Reina Sofia, 14004 Córdoba, Spain; ^9^Department of Ophthalmology, Hospital San Millán, 26006 Logroño, Spain

## Abstract

*Purpose*. To analyse variations in the anatomical and functional outcomes and in proliferative vitreoretinopathy (PVR) rate of a prospective multicentric study that was primarily designed for identification of clinical risk factors for PVR. *Methods*. 1,046 retinal detachment (RD) cases were analysed. Cases were divided into two series based upon variation in PVR rate determined by logistic regression analysis. Series 1 (S1) included RD treated during 2004-2005 (*n* = 481) and Series 2 (S2) during 2006–2008 (*n* = 565). Pre-, intra-, and postoperative characteristics were recorded. *Results*. There were few differences in the preoperative characteristics. S2 had more vitrectomies and scleral bands and fewer explants and associated cataract extractions than S1. Anatomic reattachment improved from 87.9% to 92.9% in S1 and S2, respectively, (*P* = 0.006). Visual acuity at 3 months ≥20/40 increased from 36.5% of S1 to 44.2% in S2 (*P* = 0.049). PVR rate diminished from 14.1% in S1 to 8.1% in S2 (*P* = 0.002). Centres with higher rates of PVR in S1 showed the greatest reductions in S2. *Conclusion*. An improvement in anatomical and functional outcome and PVR rate occurred in participating centres cannot be attributed to the learning curve of surgeons. We speculated that it could be an effect of their participation in the study.

## 1. Introduction

In 2005, after a retrospective case-controlled study involving five clinical centres, we developed a formula to estimate the risk of proliferative vitreoretinopathy (PVR) onset after rhegmatogenous retinal detachment (RD) surgery [1]. Even though the sensitivity and specificity values of that formula were higher than other predictive formulas [2], it was not considered appropriate for routine clinical use. Thus the Retina 1 project was designed to improve that predictive formula by increasing the sample. This project consisted of a prospective study involving 17 centres in Spain and Portugal that collected pre-, intra-, and postoperative data for consecutive, noncomplicated RDs for over 1,000 cases between 2004 and 2008.

Statistical analysis of the collected data performed after closing the recruitment period revealed an inflexion point indicating a significant reduction in PVR rate at the end of 2005. Careful analysis of the pre- and post-2005 data also showed improvements in reattachment rate which could not be attributed to differences in preoperative and intraoperative characteristics. Thus, we hypothesized that active collaborative participation of surgeons in the project might have had a positive influence in their outcomes. 

Although the main purpose of the Retina 1 project was to increase the information on the clinical characteristics which contribute to a PVR development after RD, we consider that these partial results are interesting because they emphasize some of the positive effects of any multicentric study. Therefore, the main purpose of this paper is to offer the anatomical and functional outcomes and PVR rate in one of the largest prospective series of RD and to analyze the variations during the lapse of this study.

## 2. Methods 

### 2.1. Patient Population

The protocol for this study was approved by the ethics committee of the coordinating centre (IOBA, University of Valladolid) and of each participant institution. Informed consent for RD surgery specifically included a statement of the use of data for this project. This research followed the tenets of the Declaration of Helsinki. All participating surgeons had enough experience in treating RD (minimum of 5 years of experience and at least 100 RD cases treated annually), and they were allowed to decide the surgical approach of any case according to their personal experience. A total of 69 surgeons were involved in this study. Periodically the surgeons received information regarding their partial outcomes, and they met at intervals to analyse the results.

Updated information was sent to all participants every six months, and the coordinating centre was continuously in touch with the other centres to resolve questions about how to fill in the data collection forms. The study started in October 2004 and ended in February 2008. During this period, a total of 1,046 consecutive cases of RD fulfilling the inclusion criteria were enrolled by the different centres: 14 cases in 2004 (1.24%), 467 in 2005 (44.6%), 288 in 2006 (27.5%), 259 in 2007 (24.7%), and 18 in 2008 (1.72%). 

### 2.2. Inclusion and Exclusion Criteria

All patients admitted for surgery with primary RD with a followup of 3 months were considered for inclusion. Cases with preoperative PVR grade C-1 or higher according to the Retina Society classification [3] were excluded. Cases in which the RD was due to perforating injury were also excluded.

### 2.3. Variables

A total of 83 pre-, intra-, and postoperative clinical characteristics that were gathered in the Retina 1 project were used in this paper [4, 5]. To analyse variations in these characteristics, the patients were divided into two series. Series 1 (S1) was composed of those patients treated before and throughout 2005 (*n* = 481), and Series 2 (S2) included those treated between 2006 and 2008 (*n* = 565) because of the statistically significant variation observed at the end of 2005 (see below). 

### 2.4. Statistical Analysis

Quantitative results were expressed as means ± standard deviations, and qualitative variables were described in percentages and absolute frequencies. The two series, S1 and S2, were defined according to the year in which the patients were treated based upon the results of a logistic regression model that related the rate of PVR with the year of surgery. Student's *t*-test for independent samples was used to compare each series with quantitative characteristics. Qualitative variables were compared with the *χ* ^2^ test. The Fisher exact test was used on sparse contingency tables. Statistical significance was established at the 0.05% confidence level. The statistical analysis was performed with SPSS 15.0 for Windows (SPSS Inc., Chicago, IL, USA).

## 3. Results

Logistic regression analysis showed that there was a significant relationship between the PVR rate and year of treatment (*P* = 0.008). The rates of PVR were relatively stable during 2004 and 2005. However after that year, the rate was reduced by about half. Based on this fact, we analysed patient data divided into periods S1, 2004 to 2005, and S2, 2006 to 2008. Both series were composed by an adequate number of patients for statistical analysis (S1: *n* = 481 and S2: *n* = 565). For these series, there were no significant differences in age (S1: 57.7 ± 15.1 years; S2: 57.2 ± 15.9 years), time between the beginning of symptoms and surgery (S1: 15.4 days ±27.8; S2: 18.1 ± 43.1 days), or preoperative intraocular pressure (S1: 13.3 ± 3.4 mmHg; S2: 13.3 ± 4.5 mmHg). 

Macula-on cases were more frequent in S2, 39.7%, than in S1, 33.1%, (*P* = 0.029, Table 1). Previous RD surgery was less common in S2, 8.9%, than in S1, 12.6%, although the difference was not significant (*P* = 0.06).

There were several intraoperative characteristics that distinguished the S1 and S2 series. There were more S2 cases in which a scleral band was used during pars plana vitrectomy, 59.6%, than in S1, 44,4% (*P* < 0.001, Table 2). Explants were less common in S2, 7%, than in S1, 11.6% (*P* = 0.01). Also, combined cataract extraction in S2, 7.3%, was less frequent than in S1, 12.6% (*P* = 0.004). 

Postoperative characteristics were assessed at the last follow-up visit at three months after surgery (Table 3). The reattachment rate at that time was significantly higher in S2, 92.9%, than in S1, 87.9% (*P* = 0.006). Visual acuity at 3 months was also better, ≥20/40 in 36.5% of S1 patients and 44.2% in S2 patients (*P* = 0.049). The PVR rate diminished from 14.1% in S1 patients to 8.1% in S2 patients (*P* = 0.002). 

Retrospectively, we defined three different groups of centres depending on the final PVR rates. Group 1 (G1) had a final PVR rate less than 9%, Group 2 (G2) between 10% and 13%, and Group 3 (G3) had a PVR rate higher than 13% (Table 4). There was no significant difference between S1 and S2 in G1 the PVR rates (*P* = 0.95). However, PVR S2 rates in G2 and G3 decreased significantly compared to S1 rates (*P* = 0.043 and *P* = 0.041, resp.). The number of surgeons involved in both series was 43 and although there were some variations, most of them were the same during the entire study. Further, most of them had more than 300 RD surgeries at the beginning of the project.

## 4. Discussion

Anatomical and functional results after primary noncomplicated RD surgery are now relatively stable in most of the published series, and recent papers show data that could be considered as reference results for many clinicians to analyse the quality of their practices. One such study, under the auspices of the Royal College of Ophthalmologists, consisted of a nationwide cross-sectional survey of 768 RD patients attended by 167 consultant ophthalmologists [6]. That report showed an overall reattachment rate with a single procedure was 77%. There were significant differences in reattachment rates between specialists, 82%, and nonspecialists, 71%, with a single surgery [6]. Our series (Retina 1) results were similar when compared to the specialist groups, probably because surgeries in Retina 1 were performed by experienced surgeons in all of the participating centres. The overall reattachment rate was over 88% after a single procedure and 90% at the end of followup [4, 5, 7].

As mentioned, the analysis of PVR rate showed a significant reduction after the end of 2005. Then the overall sample was divided into 2 series and there was a significant improvement in the retina reattachment and PVR rates in the S2 patients compared to the S1 patients. This was also reflected in the improvement of final visual acuity after followup. Many factors have been implicated in the improvement of outcomes after RD surgery, including the learning curve of surgeon [8, 9], changes in surgical technique from the scleral procedures to vitrectomy [5], and others. Between the S1 and S2 patient populations, there were only a few differences in the preoperative characteristics and most of the involved surgeons were the same. In the S2 series, there were more previous cases of PVR with grades A or B and previous cases of uveitis. These changes could be attributed to a better examination of the patients, because of the requisites of the inclusion criteria, and have been already reported [5]. There were also more macula-on cases in S2, although there were no differences in the evolution time between symptoms onset and surgery. In a previous partial report from the Retina 1 project, some presurgery factors were statistically related to the visual outcome [5]. Among them, the status of the macula and the existence of previous ocular surgeries other than RD influence final vision outcome. Because they were slightly more macula-on cases in S2, this fact could have influenced the final visual acuity. 

There were more cases with previous ocular surgery other than RD in S2, although the differences were not significant. Among the ocular surgeries the most frequent is cataract which is been more important because of the ageing of the population. Also more myopic eyes were treated in S2 and less PVR cases in the fellow eye were present in S2. We have not found any explanation for these findings, and we consider that they have no relevant influence on final outcomes. 

There were also some differences in surgical techniques in both series. The S2 population had more scleral bands associated with vitrectomy, more laser intervention in larger extensions of the retina, and more silicone oil injections. Other differences included fewer explants and concomitant cataract extractions in the S2, less cases of use of air as a tamponade agent in S2 and more cases of silicone oil in S2. These surgical differences for managing RD have been already reported by our group, and they do not respond to an improvement strategy but to a global change in the approaching of this disease [5]. However, the changes in the surgical approach for RD notice during the time of this project were less dramatic than those that occurred during the early years of 2000. According to our data, they did not influence the anatomical outcome comparing a short period of time of the whole prospective study (the first three years) [5]. We did not incorporate information on the vitrectomy techniques used during the study, therefore a possible bias for the use of 23G and 25G instruments on the later cases might have influenced the results [10–12]. 

Therefore, the improvement in anatomical rate and decrease in PVR rate cannot be attributed to neither the management of less complicated cases after 2005 nor the changes in the surgical approach. Because most of the participating surgeons were the same throughout the study and all were considered experienced at the beginning of the study, the improvements that we observed cannot be attributed to their learning curve. In seeking an explanation for this improvement we found no published reports suggesting that participation in collaborative studies on RD could influence the final outcomes, although several multicentric series have been published [13–16].

Participating in clinical trials contributes to the improvement in the quality of care delivered to the patients [17], and collaborative studies such as Retina 1 may share some of the common positive aspects of those studies. During the four years of the project, surgeons attended several specific meetings organized by the coordinating centre, received periodically (every 6 months) information on their personal results, and once a year they discussed the global results. These actions may have had some beneficial influence in the preoperative examination, in some changes in surgical techniques, and in the overall management of RD patients.

The prospective collection of data could have also contributed to a better examination of the eyes because it promoted the entry of more complete and accurate information into the project data collection forms. In this sense, there were differences in several clinical characteristics of the RD that could be attributed to a better preoperative examination of the patients. For instance, in the S2 population there were fewer cases of nonvisible retinal breaks, more preoperative uveitis, more familial cases of RD, and more preoperative cases of vitreous haemorrhages. 

In S1, 12.5% of the patients had previous RD surgery in other centres. That percentage fell to 8.9% in S2, which approached statistical significance. Additionally, significantly fewer patients in the S2 group had previous scleral surgery compared to the S1 group. Because most of the participant centres were integrated in the National Health Systems of Spain and Portugal, these facts can be interpreted as the recognition of the reference condition for RD treatment by the surrounding general ophthalmologists maybe as also an effect of the participation in this study. 

Visual outcome also showed a significant improvement that could be explained in part by the slight higher rate of macula-on cases in S2 and the lower PVR rate. A limitation of this study was the procedure used for judgment of anatomical reattachment. At the onset of the project, not all centres had optical coherence tomography (OCT) instrumentation. In these centres, the status of the macula and reattachment after surgery were clinically judged by indirect ophthalmoscopy and biomicroscopic examination of the fundus. If OCT had been available, some cases with poor visual acuity would likely be attributed to persistent subretinal fluid [18].

When we grouped centres according the final rates of PVR, we observed that those centres having the medium and highest rates (G2 and G3) had significant reductions in the rates after 2005. It is not the purpose of this study to analyse the reasons for these changes. Nevertheless, it is obvious that in some centres the rates at the onset of the project were inadequate elevated for a referral hospital. We hypothesize that the surgical approaches used and/or the previous examinations were not adequate. However, the final PVR rates of those centres, which were less than half of the initial ones, reinforces our hypothesis that participation in collaborative studies has a positive influence in patient's care. 

The relative stability in PVR rate in G1 centres may also support our idea that this complication has a genetic base that cannot be avoided even with a refinement of the surgical technique [19] or increasing the surgeon's experience. Thus as another conclusion of this work, a PVR rate of 6% could be considered as a reference of care quality for those centers managing RD patients.

Overall, the entire series showed a significant improvement in anatomical and functional outcomes and a clear drop in PVR rate. These improvements could be attributable to, among other factors, the continuous exchange of experiences and the feedback that surgeons received. In conclusion, large prospective clinical studies provide a huge amount of useful information and contribute to the improved care that we deliver to our patients. Such studies are difficult to organise, tedious to develop, and require a great cooperative effort. Nevertheless, the participating surgeons and their patients benefit from the sharing of outcome data, techniques, and innovations derived from the collaborative effort.

## Figures and Tables

**Table 1:** Preoperative characteristics.

Characteristic	S1		S2		*P* value
	2004-2005		2006–2008		
	*N* (%)	95% CI	*N* (%)	95% CI
Previous PVR	230 (47.8%)	43.4%–52.3%	295 (52.2%)	48.1%–56.3%	
Retinal break					0.062
Tear	348 (72.5%)	68.5%–76.5%	391 (69.3%)	65.5%–73.1%	
Hole	86 (17.9%)	14.5%–21.3%	137 (24.3%)	20.8%–27.8%	
Dialysis	6 (1.3%)	0.3%–2.2%	6 (1.1%)	0.2%–1.9%	
Giant tear	20 (4.2%)	2.4%–6%	16 (2.8%)	1.5%–4.2%	
Not visible	20 (4.2%)	2.4%–6%	14 (2.5%)	1.2%–3.8%	
Type of break					0.264
Unique	232 (50.2%)	45.7%–54.8%	293 (53.7%)	49.5%–57.8%	
Multiple	204 (44.2%)	39.6%–48.7%	228 (41.8%)	37.6%–45.9%	
Posterior	6 (1.3%)	0.3%–2.3%	11 (2%)	0.8%–3.2%	
Not visible	20 (4.3%)	2.5%–6.2%	14 (2.6%)	1.2%–3.9%	
Size of breaks (clock hours)					0.242
0-1	318 (66.9%)	62.7%–71.2%	355 (64.2%)	60.2%–68.2%	
2-3	112 (23.6%)	19.8%–27.4%	153 (27.7%)	23.9%–31.4%	
>3	25 (5.3%)	3.3%–7.3%	31 (5.6%)	3.7%–7.5%	
Not visible	20 (4.2%)	2.4%–6%	14 (2.5%)	1.2%–3.8%	
Vitreous hemorrhage	43 (9%)	6.4%–11.6%	81 (14.6%)	11.7%–17.6%	0.006
Extension RD (quadrants)					0.432
0-1	80 (16.7%)	13.4%–20%	108 (19.3%)	16%–22.5%	
2-3	341 (71.2%)	67.1%–75.2%	395 (70.4%)	66.6%–74.2%	
4	58 (12.1%)	9.2%–15%	58 (10.3%)	7.8%–12.9%	
Macula-on	158 (33.1%)	28.9%–37.3%	223 (39.7%)	35.6%–43.7%	0.029
Choroidal detachment	3 (0.6%)	0%–1.3%	11 (2%)	0.8%–3.1%	0.062
Previous uveitis	25 (5.2%)	3.2%–7.2%	58 (10.4%)	7.9%–12.9%	0.002
Previous surgery for RD	60 (12.6%)	9.6%–15.5%	50 (8.9%)	6.6%–11.3%	0.062
Previous PPV	28 (5.9%)	0%–11.9%	30 (5.4%)	0%–11.8%	0.120
Previous scleral surgery	37 (7.7%)	0.9%–14.6%	19 (3.4%)	0%–8.5%	0.017
Aphakia	161 (33.5%)	29.3%–37.8%	221 (39.2%)	35.2%–43.2%	0.062
PVD	241 (50.1%)	45.6%–54.6%	256 (45.4%)	41.3%–49.5%	0.276
Myopia	187 (50.1%)	45.1%–55.2%	244 (60.5%)	55.8%–65.3%	0.012
Ocular trauma	13 (2.7%)	1.3%–4.2%	21 (3.7%)	2.2%–5.3%	0.357
Previous ocular surgeries other than RD	198 (41.2%)	36.8%–45.6%	263 (46.6%)	42.5%–50.7%	0.080
RD in fellow eye	38 (7.9%)	5.5%–10.4%	44 (7.8%)	5.6%–10%	0.945
PVR in fellow eye	39 (8.4%)	5.9%–10.9%	18 (3.2%)	1.8%–4.7%	<0.001
Previous VA					0.051
<20/100	305 (65%)	60.7%–69.3%	325 (57.6%)	53.5%–61.7%	
20/100–20/40	59 (12.6%)	9.6%–15.6%	83 (14.7%)	11.8%–17.7%	
≥20/40	105 (22.4%)	18.7%–26.2%	156 (27.7%)	24%–31.4%	
DM	33 (7.1%)	4.8%–9.4%	52 (9.5%)	7%–11.9%	0.170
RD family history	15 (4.4%)	2.2%–6.5%	33 (7.6%)	5.1%–10.1%	0.075
PVR family history	2 (0.6%)	0%–1.5%	9 (2.2%)	0.8%–3.6%	0.123

N (%): number and percent of proliferative vitreo-retinopathy (PVR) cases; CI: confidence interval; macula-on: retinal detachment with macula attached; PPV: pars plana vitrectomy; PVD: posterior vitreous detachment; myopia: negative refractive error of any value; VA: visual acuity measured with Snellen test with pinhole if necessary; DM: diabetes mellitus.

**Table 2:** Intraoperative characteristics.

Characteristics	S1		S2		*P* value
	2004-2005		2006–2008		
	*N* (%)	95% CI	*N* (%)	95% CI
PPV	385 (80%)	76.5%–83.6%	446 (79.1%)	75.7%–82.4%	0.660
Scleral band	243 (50.6%)	46.2%–55.1%	346 (61.3%)	57.3%–65.4%	<0.001
PPV associated with band	171 (44.4%)	39.5%–49.4%	266 (59.6%)	55.1%–64.2%	<0.001
Explant	55 (11.6%)	8.7%–14.5%	39 (7%)	4.9%–9.1%	0.010
Retinopexy (cryo)	16 (3.4%)	1.8%–5.1%	28 (5.1%)	3.2%–6.9%	0.205
Cryotherapy (clock hours)					0.326
1	92 (22%)	18%–26%	115 (21%)	17.6%–24.4%	
2	42 (10%)	7.1%–12.9%	65 (11.9%)	9.2%–14.6%	
>2	16 (3.8%)	2%–5.7%	33 (6%)	4%–8%	
No	269 (64.2%)	59.6%–68.8%	335 (61.1%)	57.1%–65.2%	
External drainage	55 (11.6%)	8.7%–14.4%	63 (11.3%)	8.6%–13.9%	0.878
Tamponade agent					0.039
Air	47 (9.9%)	7.2%–12.5%	31 (5.5%)	3.6%–7.4%	
SF6	187 (39.2%)	34.8%–43.6%	226 (40.4%)	36.3%–44.4%	
C3F8	191 (40%)	35.6%–44.4%	219 (39.1%)	35.1%–43.1%	
Silicone oil	22 (4.6%)	2.7%–6.5%	37 (6.6%)	4.5%–8.7%	
No	30 (6.3%)	4.1%–8.5%	48 (8.6%)	6.3%–10.9%	
Laser (extension in clock hours)					<0.001
0–2	284 (60%)	55.6%–64.5%	244 (43.6%)	39.5%–47.7%	
3–5	86 (18.2%)	14.7%–21.7%	114 (20.4%)	17.1%–23.7%	
6–8	35 (7.4%)	5.1%–9.8%	48 (8.6%)	6.3%–10.9%	
≥9	68 (14.4%)	11.2%–17.6%	154 (27.5%)	23.8%–31.2%	
PFCL	310 (65.1%)	60.8%–69.4%	376 (66.9%)	63%–70.8%	0.543
Retinotomy	28 (5.9%)	3.8%–8%	38 (6.7%)	4.7%–8.8%	0.561
Retinectomy	8 (1.7%)	0.5%–2.9%	3 (0.5%)	0%–1.2%	0.071
Choroidal hemorrhage	6 (1.3%)	0.3%–2.3%	13 (2.3%)	1.1%–3.6%	0.212
Intravitreal hemorrhage	18 (3.8%)	2.1%–5.5%	26 (4.6%)	2.9%–6.4%	0.518
Cataract extraction associated	60 (12.6%)	9.6%–15.6%	41 (7.3%)	5.2%–9.5%	0.004
IOL	49 (10.3%)	7.6%–13.1%	33 (5.9%)	3.9%–7.8%	0.009

*N* (%): number and percent of proliferative vitreo-retinopathy cases; CI: confidence interval; PPV: pars plana vitrectomy; PFCL: perfluorocarbon liquid intraoperative use; IOL: intraocular lens implantation during surgery.

**Table 3:** Postoperative characteristics.

	S1		S2		*P* value
	2004-2005		2006–2008		
	*N* (%)	95% CI	*N* (%)	95% CI
Uveitis*	324 (67.8%)	63.6%–72%	379 (67.8%)	63.9%–71.7%	0.972
Retina reattachment rate	423 (87.9%)	85%–90.9%	525 (92.9%)	90.8%–95%	0.006
PVR rate	68 (14.1%)	11%–17.3%	46 (8.1%)	5.9%–10.4%	0.002
Choroidal detachment^†^	4 (0.8%)	0%–1.7%	17 (3%)	1.6%–4.5%	0.012
Postoperative VA					0.049
<20/100	109 (25.2%)	21.1%–29.3%	122 (22.6%)	19.1%–26.2%	
20/100–20/40	167 (38.6%)	34%–43.2%	179 (33.3%)	29.3%–37.3%	
≥20/40	158 (36.5%)	32%–41%	238 (44.2%)	40%–48.4%	

*N* (%): number and percent of proliferative vitreo-retinopathy (PVR) cases; CI: confidence interval; VA: best visual acuity corrected at the end of followup (Snellen charts); *during the first month of followup; ^†^during the first 24 h after surgery.

**Table 4:** PVR rate according to three groups of centers.

Centers (*N*)	Patients per center	PVR rate				*P* value
		S1		S2	
		2004-2005		2006–2008	
		*N* (%)	95% CI	*N* (%)	95% CI
G1 (8)	60 ± 31	10 (4.9%)	1.9%–7.9%	17 (6.1%)	3.3%–8.9%	0.956
G2 (3)	67.33 ± 33	13 (15.9%)	7.9%–23.8%	7 (5.8%)	1.6%–10%	0.043
G3 (6)	60.5 ± 58	45 (23%)	17.1%–28.8%	22 (13.2%)	8%–18.3%	0.041

PVR: proliferative vitreo-retinopathy; *N* (%): number and percent of PVR cases; CI: confidence interval; G1: <9% final PVR rate; G2: 9–13% final PVR rate; G3: ≥13 % final PVR rate.
